# Risser stages, menarche and their correlations with other growth parameters in a cohort of 3,553 Italian adolescent idiopathic scoliosis patients

**DOI:** 10.1186/1748-7161-8-S1-O13

**Published:** 2013-06-03

**Authors:** S Minnella, S Donzelli, F Zaina, S Negrini

**Affiliations:** 1 ISICO (Italian Scientific Spine Institute), Milan, Italy; 2 University of Brescia, Brescia, Italy; 3 IRCCS Don Gnocchi, Milan, Italy

## Background

Risser sign is the parameter used most often to evaluate skeletal maturity. Menarche is also claimed to be a determinant factor. In fact, the SRS criteria propose Risser sign 0 to 2, and a maximum of 2 years post-menarche, for bracing studies. But, are these parameters still valid after many years since their determination, and in all populations in various countries?

## Aim

The aim of this paper is to describe the relationship between Risser sign and other physiologic parameters in Italian patients with AIS.

## Methods

Study Design: Retrospective observational study. Population: A total of 3553 patients (2862 females, 691 males), aged between 10 and 21 years were included; each one had at least a clinical evaluation by our physicians and spinal X-rays. Methods: The two groups of females and males were separately divided into five subgroups according to Risser sign, (R0, R1, R2, R3, R4, R5), in order to analyse data of physiologic parameters (age, weight, height, BMI, menarche and two years after menarche) for each Risser level.

## Results

In females, average age of each subgroup was 13.1±1.1 (R1), 13.6±1.2 (R2), 14.6±1.2 (R3), 15.8±1.3 (R4), 17.8±1.7 (R5) with an average growth period from R1 to R5 of 4.7 years; and an average growth in height from R1 (158.8±6.9) to R5 (164.7±6.2) or 5.9 cm; as regard to weight, there was an increase of 6.3 kg from average value in R1 (47.9±8.7) to R5 (54.2±7.5); the percentage of patients who had menarche was 27% in R0, 79% in R1, 90% in R2, 97% in R3, 99% in R4, and 100% in R5; but, it's important to point out that 3% in R1, 13% in R2, 53% in R3 and 87% in R4 were two years after menarche. In males, the average growth period from R1 to R5 was also 4.7 years; but average height increased 7.2 cm; and weight increased by an average of 11.7 kg.

## Conclusion

According to our results, it's important to relate all the parameters examined for timing the stop of conservative treatment in AIS patients. Our results also show that the parameter of two years after menarche cannot be considered to determine skeletal maturity.
